# Factors associated with baseline mortality in Norwegian Atlantic salmon farming

**DOI:** 10.1038/s41598-021-93874-6

**Published:** 2021-07-19

**Authors:** Victor H. S. Oliveira, Katharine R. Dean, Lars Qviller, Carsten Kirkeby, Britt Bang Jensen

**Affiliations:** 1 Norwegian Veterinary Institute, 1433 Ås, Norway; 2 Department of Veterinary and Animal Sciences, Faculty of Health and Medical Sciences, University of Copenhagen, 1870 Frederiksberg, Denmark

**Keywords:** Ecological epidemiology, Marine biology, Ecological modelling

## Abstract

In 2019, it was estimated that more than 50 million captive Atlantic salmon in Norway died in the final stage of their production in marine cages. This mortality represents a significant economic loss for producers and a need to improve welfare for farmed salmon. Single adverse events, such as algal blooms or infectious disease outbreaks, can explain mass mortality in salmon cages. However, little is known about the production, health, or environmental factors that contribute to their baseline mortality during the sea phase. Here we conducted a retrospective study including 1627 Atlantic salmon cohorts put to sea in 2014–2019. We found that sea lice treatments were associated with Atlantic salmon mortality. In particular, the trend towards non-medicinal sea lice treatments, including thermal delousing, increases Atlantic salmon mortality in the same month the treatment is applied. There were differences in mortality among production zones. Stocking month and weight were other important factors, with the lowest mortality in smaller salmon stocked in August–October. Sea surface temperature and salinity also influenced Atlantic salmon mortality. Knowledge of what affects baseline mortality in Norwegian aquaculture can be used as part of syndromic surveillance and to inform salmon producers on farming practices that can reduce mortality.

## Introduction

High mortality of farmed salmonids results in annual deaths of tens of millions of fish across the world. Reports from some of the leading salmon producing countries (Norway, Chile, and the UK) show that the total mortality is increasing^1–3^. Norway is the world’s largest producer of Atlantic salmon (*Salmo salar* L.). The annual transfers of Atlantic salmon smolts to Norwegian coastal waters peaked at 304 million in 2018. The transfers have remained relatively stable between 2015 and 2019, with less than 2.5% variation between years^4^. Over this period, the yearly Atlantic salmon deaths has risen 27.8%, from 41.3 to 52.8 million^4^. Reducing mortality in aquaculture is crucial to ensure sustainable production. High mortality also represents major economic losses and is considered an indicator of poor fish welfare^5^. Furthermore, some of the mortality determinants that cause high mortality in farmed salmon may also threaten wild salmon, as they commonly share marine environments and are affected by the same diseases^5^.

Several studies have described regional mortality patterns in Atlantic salmon farms in Northern Europe and Chile. In Norway, previous research found large variations in mortality patterns between geographically separate areas, between years of sea transfer, and at different time points during the production cycle^2^. During the rearing period at sea, the mean mortality per month of three-quarters of the Atlantic salmon cohorts was rarely above 2%, with large variations between farms and years^2^. A proportion of monthly deaths of less than 1% in individual Norwegian farms was defined as non-extreme mortality in a study from 2018^6^. On Scottish farms, regional variation was also detected, with an average monthly mortality that varied between 0.34 and 2.81% among regions^3^. Another Scottish study reported weekly mortality below 1% in most farms (90th percentile)^7^. The average monthly mortality in the main Atlantic salmon producing regions of Chile was between 0.38 and 1.78% in 2018^1^. Building on these studies, which describe the mortality patterns in Atlantic salmon farms, the next step is to understand the main determinants of mortality that could be targeted to mitigate deaths.

Preceding mortality events, important indicators of poor welfare can be observed in fish, including behavioral changes, morphological alterations, emaciation, injuries, and other compromised physical conditions^8, 9^. Mortality is the endpoint of an adverse health condition in the fish. It is caused by a combination of environmental and host factors, and often, one or more pathogens are involved^9^. However, it is difficult to disentangle the mechanisms that play a role in mortality in general. Major infectious agents and parasites contributing to mortality in farmed Atlantic salmon include salmonid alphavirus (SAV)^10–12^, infectious salmon anemia virus (ISAV)^13, 14^, infectious pancreatic necrosis virus (IPNV)^15, 16^, piscine myocarditis virus (PMCV)^17, 18^, *Moritella viscosa*^19^*, Renibacterium salmoninarum*^20^, sea lice (*Lepeophtheirus salmonis* and *Caligus* spp.)^21–23^, and *Paramoeba perurans*^24^. Coinfections can occur, and those involving viruses (e.g. IPNV and SAV) and sea lice infestations or other infections (e.g. with ISAV and *M. viscosa*) are associated with higher mortality^16, 25, 26^.

Environmental factors, including algal blooms and changes in temperature and salinity, are also known for their potential influence on mortality^27–30^. For example, high sea surface temperature and salinity (> 12 °C and > 12‰) can increase *L. salmonis* growth and survival rates^31, 32^, with effects on Atlantic salmon infestation by the parasite. Similarly, environmental conditions can influence the growth of different species of pathogenic bacteria and toxin-producing algae that are harmful towards Atlantic salmon^19, 29, 30, 33^. In contrast, higher temperatures appear associated with lowered IPNV prevalence within farms^16^.

The susceptibility of the farmed Atlantic salmon to diseases associated with higher mortality may vary throughout the production cycle and according to stocking conditions. Detection of IPNV usually occurs during the first 3 months after the salmon transfer to sea^15, 16^, while PMCV affects larger salmon (> 2 kg), which have spent longer periods at sea^17, 18^. Both SAV and *R. salmoninarum* have been detected in salmon across a wide range of ages^20, 34, 35^. A factor that can also contribute to greater mortality risk is the lack of vaccinations, for example against SAV^36^ and *M. viscosa*^37^. Management practices adopted by farmers for controlling sea lice in Atlantic salmon constitute another important challenge, particularly in high-density farming areas^38^. Sea lice treatments using hydrogen peroxide (H_2_O_2_) baths can be toxic to Atlantic salmon^39^. The development and spread of resistance in sea lice towards medicinal treatments (e.g. emamectin benzoate, organophosphates, and pyrethroids) and H_2_O_2_ bath treatments adds to the burden of the parasite. This has shifted management strategies towards the use of so-called “non-medicinal” treatments, with sea lice removal by warm water, flushing or brushing^6, 40–42^. These treatments are considered responsible for inducing trauma and increased mortality in Atlantic salmon^41, 43–45^.

To date, most investigations into the mortality determinants in Atlantic salmon farming have focused on specific factors presumed to be associated with extreme mortality, with little research on the factors associated with underlying baseline mortalities during production. Here, the baseline mortality refers to the mortality at a “normal” (or expected) level, not including extraordinary events associated with increased mortality, such as algae blooms or infectious disease outbreaks. The objective of this study was to identify the determinants of the baseline mortality in the Norwegian population of farmed Atlantic salmon and to quantify their effects, using routinely collected environmental, fish health, and production data.

## Results

### Description of baseline Atlantic salmon mortality

**Figure 1:**
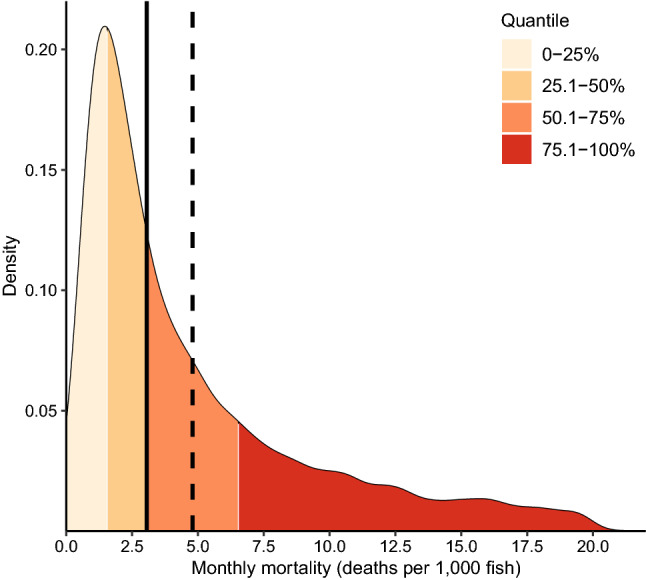
Summaries of monthly mortality distribution between 2014 and 2019 in Norwegian Atlantic salmon farms. The smoothed line is the kernel density estimate, quantiles are represented by colors, the median by the straight line and the mean by the dashed line. This figure was generated using the Tidyverse^46^ package in R^47^.

**Figure 2:**
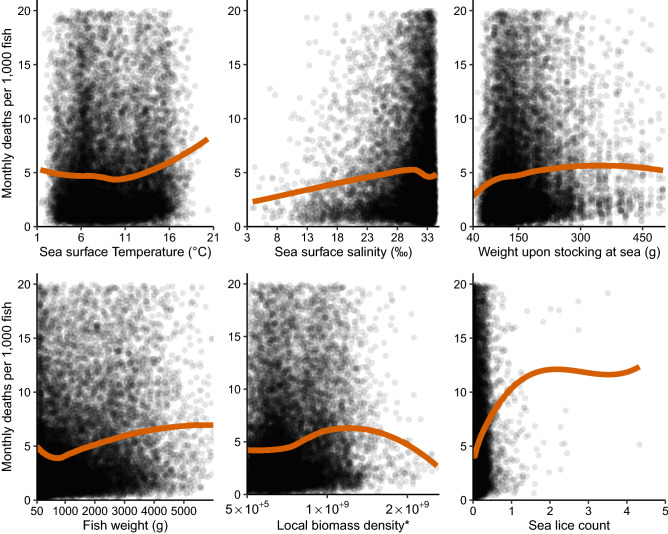
Scatter plots with fitted local polynomial regression (LOESS) curves of mortality versus continuous explanatory variables. *The local biomass density (LBD) of a farm in a certain month is a summarized record of Atlantic salmon biomass (i.e. number of fish multiplied by mean weight) calculated using data from neighboring farms located up to 40 km seaway distance. For full details on LBD calculations, we refer to Jansen et al.^38^. This figure was generated using the Tidyverse^46^ and gridExtra^48^ packages in R^47^.

Our study population consisted of Atlantic salmon put to sea from 1627 cohorts produced on 642 different farms. Among the 14,280 records of the analyzed dataset, the mean number of fish at risk per month was 838,228 (median = 777,328; interquartile range [IQR] = 493,233–1,108,106). The mean monthly number of dead fish was 4.8 deaths per 1,000 fish (median = 3.05; IQR = 1.58–6.53) (Fig. 1). We present the descriptive results of salmon mortalities and its putative determinants in plots (Fig. 2) and in data summaries (Table 1).

**Table 1:** Descriptive statistics of categorical explanatory variables versus mortality.

Variable	Category	n	Monthly deaths per 1000 fish		
			Mean	Median	IQR^a^
Production zone^b^	1	332	4.1	2.26	1.39–5.69
	2	939	5.36	3.76	1.79–7.99
	3	2884	5.71	4.02	1.80–8.44
	4	2077	5.2	3.47	1.68–7.32
	5	598	4.78	3.15	1.95–6.02
	6	1870	4.61	2.83	1.54–6.06
	7	498	3.6	2.15	1.19–4.39
	8	1270	3.8	2.37	1.30–4.74
	9	1136	3.66	2.29	1.20–4.59
	10	692	3.73	2.38	1.29–4.71
	11	540	5.08	3.37	1.79–7.10
	12 and 13^c^	1444	5.03	3.63	1.97–6.57
Month of 1st stocking at sea	Mar	1158	5.48	3.56	1.77–8.07
	Apr	2561	5.29	3.60	1.80–7.41
	May	2173	4.7	3.07	1.63–6.22
	Jun	690	5.0	3.24	1.76–7.23
	Jul	1046	4.88	3.37	1.78–6.57
	Aug	2362	4.43	2.85	1.45–5.97
	Sep	2607	4.51	2.51	1.31–6.06
	Oct	965	4.24	2.58	1.41–5.47
	Nov	298	5.18	3.79	1.49–7.33
	Dec, Jan and Feb^c^	420	4.65	3.42	1.79–6.21
H_2_O_2_ or medicinal treatments^d^	Not treated	11,162	4.42	2.77	1.48–5.84
	1 time per month	2189	5.94	4.35	2.15–8.61
	≥ 2 times per month^c^	929	6.63	4.97	2.21–10.0
Non-medicinal treatments^e^	Not treated	13,039	4.48	2.81	1.50–5.97
	1 time per month	920	7.72	6.68	3.72–10.8
	≥ 2 times per month^c^	321	9.2	8.66	4.58–13.3

^a^*IQR* interquartile range.

^b^Production zones delimited in Fig. 6.

^c^Merged category due to few number of observations for one of them.

^d^Bath treatments using H_2_O_2_ or medicinal compounds, such as azamethiphos and pyrethroids.

^e^Removal of sea lice usually by flushing or brushing, warm water or freshwater baths.

### Model fitting and diagnostics

Models with interactions did not converge and we excluded them from our analysis. The interactions also caused high collinearity problems, confirmed by a variance inflation factor higher than 10. We log-transformed the variable local biomass density for convergence but this variable was later dropped from our model due to lack of significance. We also eliminated the variable for sea lice infestation from models because its inclusion caused a lack of uniformity in the distribution of the standardized residuals. In our final model, the variables sea surface temperature (SST) and fish weight were polynomial terms of the second order. This was due to curvilinear patterns observed in the scatter plots of these variables against the outcome; in addition, we detected considerable improvements of the model fit by including second order polynomial terms. The final multivariable model had eight significant variables (*p* < 0.05) associated with mortality (see Supplementary Table S1 online), which were: SST, sea surface salinity (SSS), production zone, initial weight upon stocking at sea, month of first stocking at sea, fish weight, H_2_O_2_ or medicinal sea lice treatments, and non-medicinal sea lice treatments. There was a borderline difference between this final model, Akaike information criterion (AIC) = 257,353, and a model without the variable initial weight upon stocking at sea (AIC = 257,355.4). The final model intra-class correlation coefficient (ICC) was 0.154. The variable SSS showed potential confounding effects with the production zone 1 and initial weight upon stocking at sea. We did not detect model fit issues from the residual plots (see Supplementary Fig. S1 online).

### Determinants of baseline salmon mortality

**Figure 3:**
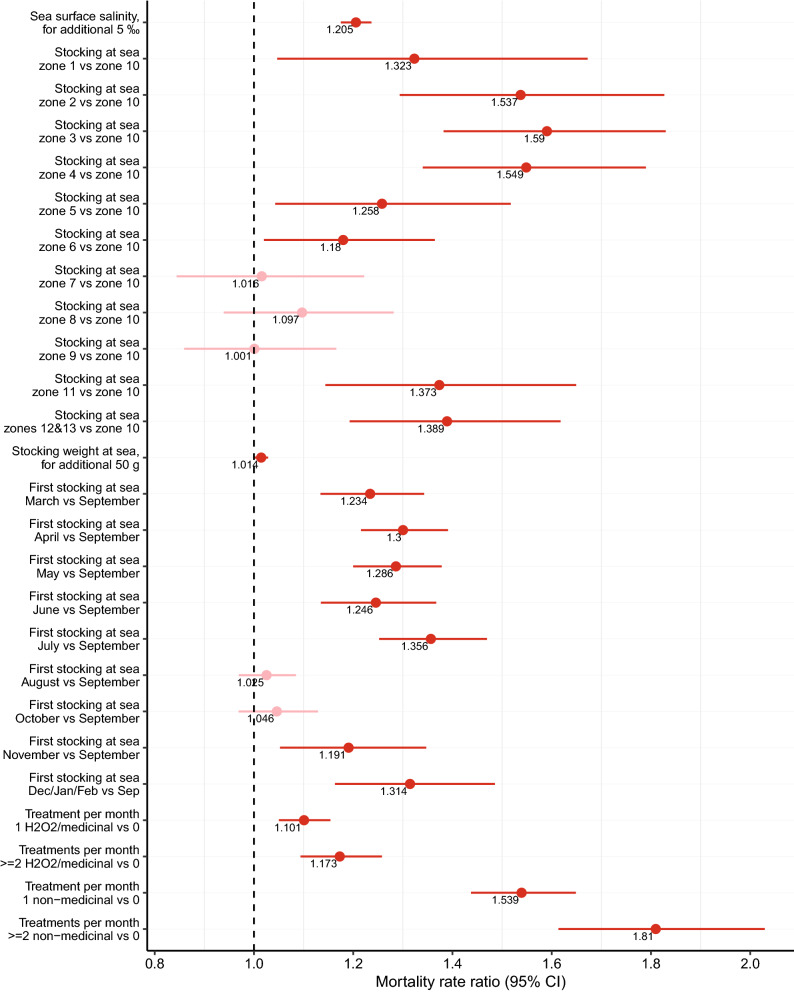
Mortality rate ratio plot with the determinants of baseline mortality in farmed Atlantic salmon. Results of other determinants added as polynomial terms in our mortality model are shown in Figs. 4 and 5. This figure was generated using the Tidyverse^46^ package in R^47^.

**Figure 4:**
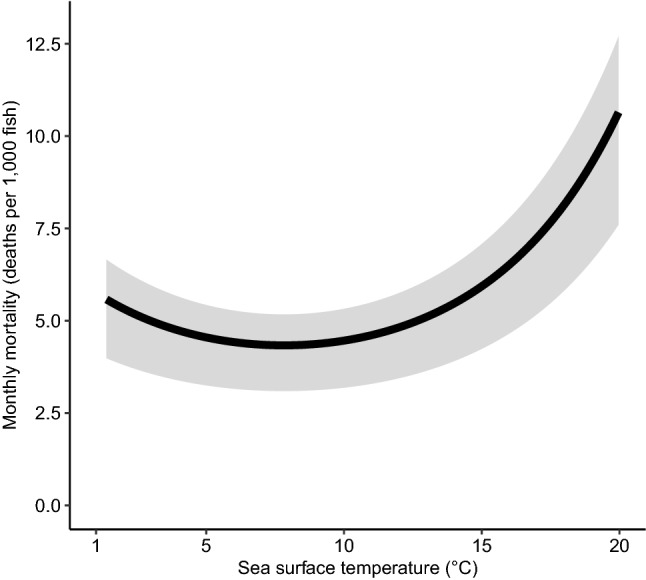
Atlantic salmon mortality predictions from the final model based on sea surface temperature. The lines and shaded areas in the plots show the mean and interquartile range of predicted values, respectively. This figure was generated using the Tidyverse^46^ package in R^47^.

**Figure 5:**
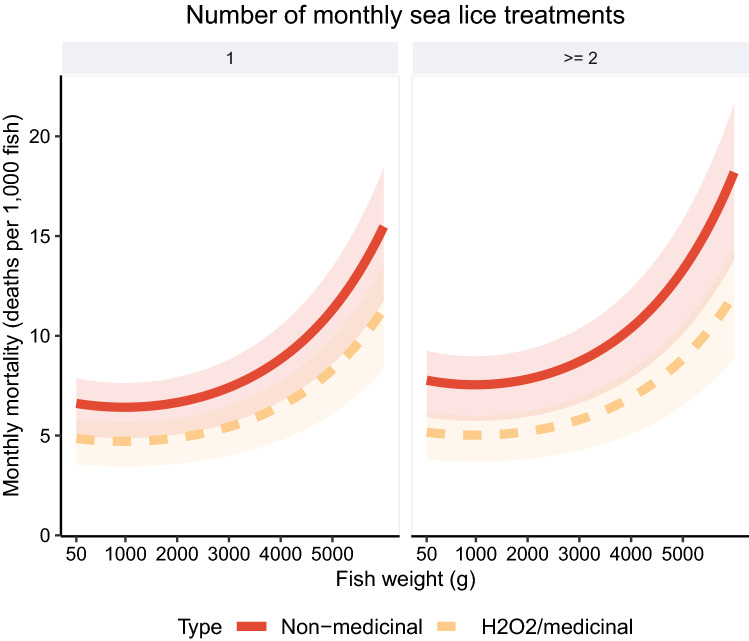
Atlantic salmon mortality predictions from the final model based on fish weight and number of sea lice treatments per month. The lines and shaded areas in the plots show the mean and interquartile range of predicted values, respectively. The different colors represent two types of sea lice treatments for comparisons. One is bath treatments using H_2_O_2_ or medicinal compounds, such as azamethiphos and pyrethroids. The other is non-medicinal treatments with removal of sea lice by flushing or brushing, warm water or freshwater baths. This figure was generated using the Tidyverse^46^ package in R^47^.

Results from the final model of baseline mortality in farmed salmon are presented in a plot with mortality rate ratios (Fig. 3) and plots of predicted mortalities (Figs. 4, 5). For the environmental variables, we predicted higher mortality when the SST was below 5 and above 10 °C (Fig. 4). Higher SSS had an adverse effect on mortality (Fig. 3); for each additional 5‰ increase in salinity, we estimated that mortality increased by approximately 20%. The production zones with the highest mortality were 2, 3, and 4 (Fig. 3). These zones had approximately 1.5 times higher mortality compared to the production zone 10, which was the least problematic region in terms of mortality. Salmon transferred to sea in July had the highest mortality compared to salmon transferred to sea in the late summer and fall (Fig. 3). Higher weight at the time of first stocking at sea had a small, negative impact on mortality (Fig. 3). The estimated mortality was high for the smallest fish, but decreased until around 500 g, when the mortality started to increase. We found a pronounced increase in the predicted number of deaths in salmon heavier than 2000 g (Fig. 5). The most detrimental factor among all the mortality determinants was the use of non-medicinal treatments for sea lice. When salmon were subjected to such treatments, the mortality was almost double than without them. The H_2_O_2_ or medicinal sea lice treatments also influenced the mortality, but to a lesser extent (Fig. 5).

## Discussion

In this study, we identified and quantified important factors that contribute to mortality on Norwegian salmon farms. We explored salmon mortality in a different way compared to previous research, as our focus was on the baseline mortality (≤ 2%). This provided a deeper understanding of what determines mortality beyond specific adverse events associated with extreme numbers of deaths. It also supplies the farmers with a way of benchmarking the performance of their farms in relation to national or regional averages. Our study utilized extensive longitudinal data and made use of discrete death counts. The advantage of this approach is that it was possible to adjust mortality predictions to the number of fish at risk of death per month. Furthermore, we relied on data that is routinely collected in Norway, which offers the opportunity to monitor excess deaths on farms by comparing the observed deaths to the expected deaths in a baseline situation.

Previous studies of mortality on salmon farms have used different outcome variables, which can make it difficult to compare results. An earlier study of Norwegian farms modelled the probability of fish deaths^2^. A Scottish study modelled mortality using the proportion of fish biomass lost within a month as the outcome^3^. Using biomass data as the outcome can be a potential source of uncertainty because of variations in the records depending on the stage of the production cycle, as larger fish contribute more to the losses than smaller ones. We calculated mortality based specifically on the counted deaths, and other attributed reasons for fish losses, such as discarded and escaped fish, were excluded. These excluded categories accounted for approximately 10–15% of the salmon losses between 2015 and 2019 in Norwegian aquaculture^4^. However, using death counts can have some limitations. For example, it was necessary to exclude records from our analyzed dataset due to inconsistencies in the numbers of added and removed fish on some of the farms between consecutive months. These inconsistencies are often related to farm management, such as moving or splitting the fish or having multiple stocking or slaughtering events, which can generate unmatched counts. Access to the detailed production data from the farmers could resolve some of the inconsistencies, as farmers usually keep daily information on movements and data counts. However, such access is often only granted to a subset of the population and, therefore, could introduce biases relating to specific management practices and willingness to provide higher resolution data.

Overall, we found factors related to production, health, and the environment that were determinants of mortality. In terms of production, fish weight can be interpreted as an indicator of the temporal pattern of mortality throughout the production cycle. Hence, our results correspond with others^2, 49^, who also found high mortality during the initial 1–3 months at sea (generally when fish grow from 50 to 500 g) with a subsequent drop that is followed by a rising trend in mortality until the end of the production. It is reasonable to assume that the higher mortality in older salmon is a consequence of longer periods of fish exposure to mortality determinants. For example, sea lice treatments happens more frequently in the second year in sea. In the initial period at sea, the weekly mortality reached values above 4%. This could be attributed to so-called “failed smolts”, when transformation from parr to smolt is not complete^7, 50^. In the early stages of salmon adaptation to seawater, hypo-osmoregulatory disturbance, reduced feed intake, and a potential down-regulation of immune responses may increase salmon mortality^51, 52^.

Other production related factors that showed effects on mortality were the month and weight at first stocking. Salmon transfers to sea occur throughout the year using smolts from season (or “S1”) and off-season (or "S0") production, which are distinct in terms of the transfer age. In general, the S1 smolts are more than 1 year old and transferred during spring, whereas the S0 can be transferred at a younger age during autumn^53^. Our study revealed a lower mortality when the transfers occurred between August (late summer) and October (autumn), which is comparable with previous research that found lower mortality of S0 smolts in the 90 days post-transfer period comparing to S1 smolts^54^. In addition to the stocking month, the starting weight of fish seemed to have adversely affected mortality the higher it was. This result is in agreement with others^55^, who suggested that potential desmoltification of larger fish could be an issue. The transfer of smolts to sea generally occurs when fish weigh less than 150 g^56^. By contrast, the transfer of larger smolts to sea can be defended as a way to minimize exposure of the host to sea lice and other infectious agents during their life cycle. There are now protocols for land-based systems to rear larger salmon (up to 450 g) prior to the sea transfer^28^. However, we did not detect a protective effect from stocking larger smolt and the observed effect of variations in the initial weight for the transfer were generally small in our study.

Apart from management factors, we found health-related factors that were associated with mortality including production zone and the number of sea lice treatments. Production zone can be a proxy for several factors, including those not retained in the final mortality model (i.e. local biomass density and sea lice infestation), but overall they have been shown to be related to the prevalence of infectious diseases. We found that the highest mortality was in the zones 2, 3 and 4, which are the zones that have traditionally had problems with sea lice infestations, and diseases such as cardiomyopathy syndrome (CMS) and pancreatic disease (PD)^4, 5, 57^. For example, the estimated weekly louse larvae production per fish in zones 2, 3, and 4 were the highest and more than twice that estimated for most of the other zones^4^. The high sea lice infestation level can also indicate where delousing treatments associated with mortality are undertaken more frequently. It has been reported that fish groups from farms in southern and western Norwegian coastal waters, corresponding to the high mortality zones, were at higher risk of developing clinical CMS^57^. Regarding PD, there are two subtypes of the virus causing the disease in Norway (SAV 2 and SAV 3), with SAV3 being associated with higher mortality^12^. The SAV is endemic in just under half of the production zones, but almost all SAV 3 cases in Norway are detected within the zones 2, 3 and 4^4^.

The use of baths with H_2_O_2_ or medicinal compounds as well as non-medicinal treatments against sea lice also contributed considerably to mortality. Overall, there was a decrease in the number of sea lice treatments from 2014 (n = 3000) to 2019 (n = 2678), despite of a slight increase in the number of fish harvested^4^. Notably, this has been accompanied by a dramatic shift away from medicinal bath treatments, driven by the development of resistance in sea lice^6, 40–42^. In 2014, most of the treatments were medicinal baths and H_2_O_2_ (60% and 34%, respectively), whereas in 2019 the majority were thermal and mechanical delousing (54% and 27%, respectively)^4^. Thus, we attribute the decline in the total number of sea lice treatments to the shift away from less effective bath treatments towards non-medicinal technologies which have become more effective in the last years^6^. Although bath treatments are less widely used, they are still an important risk factor for mortality. Activities associated with bath treatments, such as crowding of fish as well as loading and unloading of well boats, can induce a stress response in fish leading to increased mortality^58^. Studies argue that mortality solely related to medicinal applications does not occur following dosage recommendations^59, 60^. However, with H_2_O_2_ there was increased mortality when baths were performed above 10 °C^39, 61^, which is a commonly registered temperature in production sites.

We found that the non-medicinal treatments were associated with the highest mortality in comparison with the other determinants. This is consistent with previous research that found mortality increases from 1 month to the next were mostly due to thermal and mechanical delousing compared with the other delousing methods used on Norwegian farms^6^. A study has quantified the effects of different delousing treatments on mortality, and found that 790 fish are expected to die within the first two weeks following a thermal treatment, as compared to 928 from mechanical treatments and 146 from medicinal treatments (based on sea cages with an average of 150,000 salmon and stocked in 2017)^41^. The negative impacts of the non-medicinal treatments on other fish welfare indicators have also raised concerns. There was an association between thermal delousing and snout and fin injuries in salmon^43, 44^. Furthermore, salmon exposed to warm water at 28 °C for 10 s probably suffered pain as indicated by behavioral changes, which is less time than the thermal treatments at 28–34 °C for 30 s used in farms^62^. An unusual increase in skin bleeding and scale losses were observed in salmon treated by mechanical delousing^43^.

Non-medicinal treatments might be the only option for some farmers to adhere to regulations regarding control of sea lice, despite the understanding of their negative effects on fish welfare and mortality. Quantifying these effects has important implications for the use and development of other management strategies against sea lice that are probably less detrimental to salmon. This includes the preventative approach of sea cages with “skirts”, which are already commonly used on some commercial farms. The “skirts” are manufactured with materials that minimize salmon contact with sea lice on the sea surface^63^. A meta-analysis study found just above 50% median reduction in sea lice infestation density related to the sea cages with “skirts”^64^. Biological control of sea lice using cleaner fish (e.g. Atlantic lumpfish (*Cyclopterus lumpus*), ballan wrasse (*Labrus bergylta*), goldsinny wrasse (*Ctenolabrus rupestris*) and corkwing wrasse (*Symphodus melops*)) that eat the parasite attached to salmon is another management strategy adopted by some farmers. It is estimated that 49.1 million cleaner fish were placed in sea cages with Atlantic salmon in 2019^4^. With regards to the success of cleaner fish in reducing sea lice infestation, there seem to be variations based on studies conducted in commercial scale farms^65^. Co-stocking cleaner fish with salmon failed to reduce sea lice counts^66^, whereas others found high reduction (60–100%) in adult female lice counts compared with sea cages without cleaner fish^67^. It is noteworthy that the negative impacts of using cleaner fish, especially wild-caught wrasse, has been debated, due to the potential introduction and exchange of pathogens between different fish species stocked in the same sea cages^4, 68^. In addition, the mortality of cleaner fish during their time spent in sea cages with the Atlantic salmon is extremely high. According to a survey performed in 2019, the median mortality of cleaner fish across Norway was 42%, and so, there is a welfare issue with using these fish^4^.

Finally, temperature and salinity were the variables considered to evaluate exposure of salmon to adverse environmental conditions. Extremely high (> 22 °C) or low (< 2 °C) temperatures can be lethal to salmon^69^_._ We rarely observed these extreme temperatures in our study. However, we still found that temperature had a non-linear effect on mortality, and temperatures outside the optimal range of 5 and 10 °C were associated with increased mortality. When it comes to salinity, the recorded related data was often close to 33‰ and values lower than that generally resulted in lower mortality. Prior studies on farmed Atlantic salmon focused on the influence of temperature and salinity in early post-smolts^27, 55, 69, 70^. In the days after transfer, the osmoregulation of post-smolts in tanks with a salinity of 33‰ is likely to be impaired at both low temperatures (4.1 °C and 4.3 °C)^55, 70^ and high temperatures (14.3 °C)^70^, resulting in negative impacts on mortality. It is possible that absolute temperature and salinity do not necessarily affect salmon mortality directly. For example, salmon may be more sensitive to abrupt changes in temperature and respond differently to such changes depending on its life stage^62, 69^, which we did not consider in our study.

A report from 2019 shows how an increase in mortality for larger fish happened during the period from 2009 to 2016, and this increase was greater at high temperatures^71^. We also cannot exclude the indirect effects of temperature and salinity on salmon mortality through their influence on the development of harmful algae^29, 30^ and pathogens^16, 19, 33^, including *L. salmonis*^31, 32^. A limitation to our study was that we were not able to assess other environmental variables due to a lack of nation-wide data. As pointed out by others^7, 72^, reduced oxygen levels, strong water currents, predation, and algal blooms can have consequences for fish welfare and mortality. Although the explored environmental factors cannot be controlled in salmon production sea cages, their association with fish health is useful to monitor mortality.

The baseline mortality model raises the possibility for developing a syndromic surveillance system for farmed salmon. Syndromic surveillance uses data regularly updated to detect possible deviations from typical records that could be related to health problems in a population^73^. Such deviations demanding further attention might be detected in a timely manner using predictions from models that take into account the spatial-temporal patterns of mortality as well as its determinants for retrospective data analysis. Hence, interventions aiming to decrease salmon mortality might be feasible. The use of mortality data for syndromic surveillance is promising and has been explored mainly for cattle^73, 74^.

## Conclusions

This study has shown how some environmental, geographical, production and health determinants explain the baseline mortality of farmed salmon. The multivariable regression analysis revealed that the sea surface temperature and salinity were important environmental mortality determinants. There was higher mortality in some zones associated with more disease occurrence. Although increased mortality appeared in the first months after sea transfer of salmon, this problem was more noticeable later in the salmon production cycle. Several of the mortality determinants were connected to the intensive salmon production system. This included the period of the year when fish was first stocked at sea and practices undertaken to tackle major salmon diseases, especially sea lice treatments. There were considerable effects of applied treatments against sea lice on mortality using baths with H_2_O_2_ or medicinal compounds as well as non-medicinal delousing, the latter being more detrimental. It is promising to use the regularly reported data for mortality predictions. Thus, results of this study also offer the possibility to monitor mortality patterns for early detection of health problems in salmonids.

## Methods

### Study design and population

The production cycle of Norwegian salmon farming has a freshwater phase followed by a seawater phase. This study included only the seawater phase, from the time smolts are stocked at sea until they are slaughtered. The first stocking of smolts at sea is usually unevenly dispersed throughout the year, but smolts are more frequently stocked during spring and autumn months. Smolts are typically moved to sea cages when they are between 6 and 12 months old. The salmon are slaughtered after approximately 14 to 18 months of life at sea. Longer periods of life at sea are possible depending on fish growth rates and the farmers’ management preferences. When all cages on a farm are emptied, a minimum fallowing period of 2 months is obligatory in Norway before stocking a new fish cohort in the farm.

**Figure 6:**
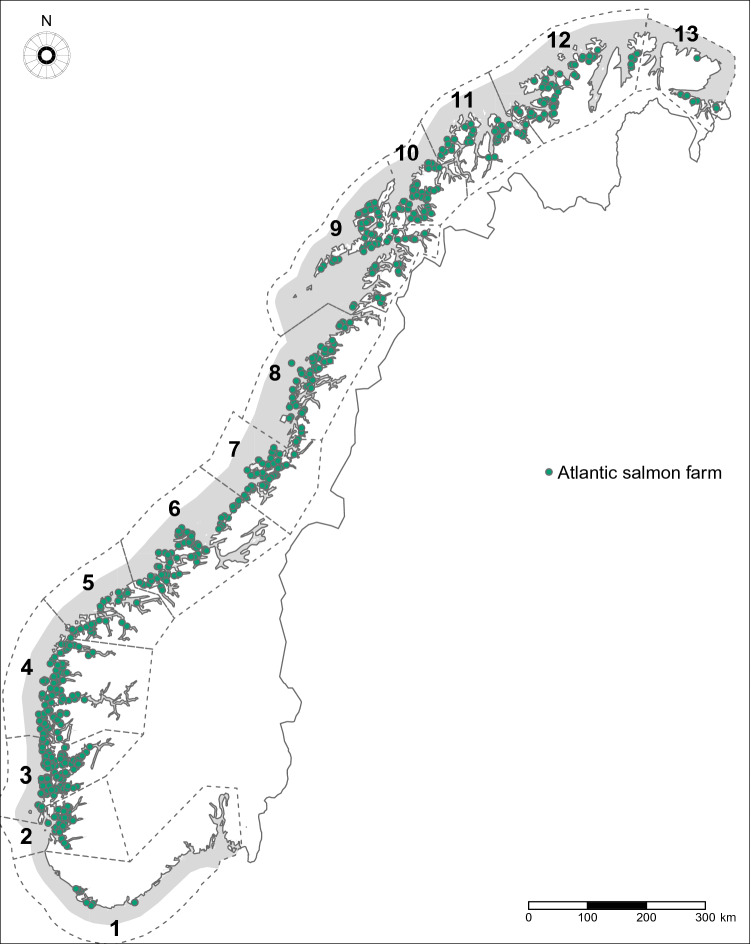
Geographical location of Atlantic salmon farms and production zones in Norway. The map shows all commercial farms for the purpose of food production registered between January 2014 and December 2019. The regions delimited by dashed lines represent the production zones 1–13. This map was generated in R, using the R packages sf^78^ (version 0.9-7; https://cran.r-project.org/web/packages/sf/sf.pdf) and tmap^79^ (version 3.3-1; https://cran.r-project.org/web/packages/tmap/tmap.pdf).

Our target population was Atlantic salmon (*Salmo salar* L.) from commercial farms in Norway, farmed for the purpose of food production. Figure 6 shows registered farms (n = 717) in the national database during the period of this study, between January 2014 and December 2019. During this period, the number of active farms per month ranged from 271 to 421. These farms are distributed across 13 distinct production zones along all the Norwegian coast, established by regulations^65^. Common characteristics of farms located within a zone includes their water current connectivity and geographical location. The zones have been used for management decisions, for example, strategies towards minimizing the spread of sea lice^75–77^. The density of farms in each of the zones varies considerably.

**Figure 7:**
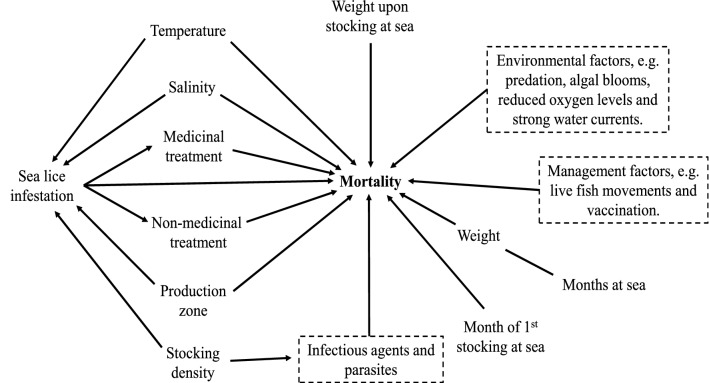
Directed acyclic graph (DAG) showing the putative determinants of baseline mortality in farmed Atlantic salmon. Dashed boxes represent unobserved factors.

We conducted a retrospective study using farm-level data. We used the count of dead fish for statistical inferences and predictions. Based on existing literature and discussions among the authors, we hypothesized determinants and their putative association with salmon mortality and summarized this in a directed acyclic graph (DAG) (Fig. 7).

### Data sources and processing

We gathered environmental, fish health and production data. Farmers reported monthly data to the Norwegian Directorate of Fisheries, which were: stocking month, fish counts, deaths and other types of losses, and mean fish weight. Because there were few records from farms located in zone 13, we grouped data from zones 12 and 13 together. For some of the data, farmers reported to the Norwegian Food Safety Authority on weekly basis. Sea surface temperature is one of them. Another one was the sea lice infestation level data, which was based on the mean number of mature female sea lice. A minimum of 10 sampled fish from half of the cages, but no less than 2 cages per farm were considered for the sea lice counts reported between January 2012 and March 2017. From March 2017 onwards, sea lice counts were made across all cages. Sea lice treatments are required if the infestation level exceeds a threshold of 0.5 lice per salmon^80, 81^. This threshold was reduced to 0.2 during spring after the regulations from 2017, due to the threat of sea lice to wild salmon smolt in their migration period to sea. The number of treatments applied to control sea lice was also acquired weekly and refers to two types. The first is bath treatments using H_2_O_2_ or medicinal compounds, usually azamethiphos, cypermethrin, and deltamethrin. The other is non-medicinal treatments, which included the removal of sea lice by flushing or brushing (mechanical delousing), warm water (thermal delousing) or freshwater baths. Farmers can use a web portal for data entry. Most commonly, commercial software is used for transferring data to the portal. We have access to those data through the Norwegian Food Safety Authority (not open access), but some of the data is available online (https://www.barentswatch.no/en/fishhealth/). Daily records of sea surface salinity (SSS) were provided by the Institute of Marine Research, and were estimated from the NorKyst800 hydrodynamic ocean model^82^. The SSS in a farm on a day matched with the SSS estimated in the closest geographic coordinates.

We processed and analyzed the data using R statistical software version 4.0.2^47^. Farm registration numbers and geocoded coordinates were unique identifiers in the datasets, available online (www.fiskeridir.no). We used the tidyverse collection of packages to manage and visualize data^46^; the packages sf and tmap for generating a map with the study farms^78, 79^ and the ncdf4 package^83^ for reading the SSS data from NetCDF format. Data inputs on a weekly and daily basis were converted into monthly records using the mean of values related to a month. An overview of variables and their scale used in this study can be found as Supplementary Table S2.

The complete dataset had 26,285 monthly records from salmon cohorts before we applied exclusion criteria. We excluded data with inconsistencies when, in the same month, the number of deaths was larger than the fish count (number of monthly records (*n*) = 16). In addition, we disregarded records where the number of counted fish in a month did not match with the expected number after the removal of fish losses from the previous month (*n* = 7003). There was also an exclusion of records from cohorts where fish had a mean initial start weight higher than 500 g (*n* = 1394). The listed inconsistencies could be related to farm management practices, including live fish movements between cages or farms, splitting of production cohorts, and multiple stocking or slaughtering events. In such cases, it was likely that fish had not spent the whole production cycle at sea on the same farm. Therefore, we would not have reliable data regarding their retrospective exposure to putative mortality determinants. We further excluded records that did not represent salmon from a typical farm for commercial purposes, i.e. records from months when fish had more than 24 months spent at sea (*n* = 26) and with fish weighing more than 6 kg (*n* = 56). Because our objective was to model baseline mortality, we excluded records when the monthly mortality rate (see next section) was higher than 2% (*n* = 3510). We set this limit for baseline mortality based on reports that described the mortality patterns in salmon aquaculture in Norway^2, 6^ and elsewhere^1, 3, 7^. Using Norwegian reports to illustrate^2, 71^, the mean monthly mortality was generally below 2%, in three-quarters of the salmon cohorts produced in sea cages between 2014 and 2018^2^. The final dataset for analysis comprised 14,280 records after the exclusions, which corresponded to 54.3% of the data obtained during the study period. This data referred to approximately 90% (642/717) of the farms in our target population.

### Statistical analysis

Fish mortality is described as mortality rates in this paper. We calculated the monthly mortality rate (MR) using the following equation, with an approximate calculation of the number of fish units at risk for the established period of time in the denominator^84^:

1 $$MR = \frac{{deaths}}{{\left( {start - \frac{{deaths}}{2} - \frac{{wth}}{2} + \frac{{add}}{2}} \right)*time}},$$

where *deaths* is the number of dead fish in a month; *start* is the number of fish at risk at the beginning of the month; *wth* is the number of withdrawn fish from the population; *add* is the number of added fish to the population; and *time* is one month.

For descriptive purposes, we produced plots with fitted local polynomial regression (LOESS) curves and tables with the calculated mortality rates and its distribution over the variables included in the DAG.

We implemented generalized linear mixed models using the glmmTMB package^85^. Our outcome was the number of dead fish, which we modelled using negative binomial regressions, suitable for count data. The negative binomial regression also accounted for overdispersion in the data, which we confirmed after fitting quasi-poisson models that revealed large dispersion parameters. We log transformed the number of fish at risk every month [the denominator in Eq. (1)] and used it as an offset term in the right hand side of models. We included the putative mortality determinants as explanatory variables in the model as fixed effects. We also included farm as a random effect to account for repeated measures. The model is defined as follows:

2 $${Y}_{i}=NB({\mu }_{i},k)$$

3 $$E\left({Y}_{i}\right)={\mu }_{i}$$

4 $$var\left( {Y_{i} } \right) = \mu _{i} + \mu _{i}^{2} /k$$

5 $${\text{log}}\left( {\mu _{i} } \right) = \beta _{0} + X_{i} \beta + \log \left( {t_{i} } \right) + \gamma _{{farm}} + \varepsilon _{i}$$

6 $${\gamma }_{farm}\sim N\left(0,{\sigma }_{farm}^{2}\right),$$

where ${Y}_{i}$ is the number of dead fish per month in farm *i*, ${\mu }_{i}$ is the expected number of deaths, $k$ is the dispersion parameter, ${\beta }_{0}$ is the intercept, ${X}_{i}$ is the matrix of variables, $\beta$ is the vector of regression coefficients, ${t}_{i}$ is the fish-time units at risk per month in farm *i*, and ${\gamma }_{farm}$ is the random effect of farm.

We began model selection with a global model including the explanatory variables described in Supplementary Table S2 online. We included interactions and polynomial terms in the model if applicable. In each step, we eliminated the variable with the weakest association with the outcome. The variables retained had an association with the outcome at a 5% significance level based on results of likelihood ratio tests. Furthermore, we calculated the variance inflation factor of the variables to check for collinearity problems. We selected the final model based on the Akaike information criterion. We assessed potential model confounders by excluding variables from the final model one by one and refitting the model. We considered a variable to have confounding effects if after its exclusion there was a change greater than 20% in at least one of the regression coefficients when comparing it to the final model results. The potential confounders were kept in the final model. Model results consisted of estimated regression coefficients and 95% profile likelihood confidence intervals. We estimated the contribution of the random effect to the model by computing the intra-class correlation coefficient using the performance package^86^. We visualized the model results using plots of the predicted number of fish deaths versus generated values for the variables in our final model.

For model validation, we ran 1000 simulations to produce standardized residuals using the DHARMa package^87^. We then visually inspected the residuals plotted against the fitted values, and against each of the explanatory variables.

## Supplementary Information

Supplementary Information.

## Data Availability

Part of the data used is routinely updated and publicly available online (www.barentswatch.no/en/fishhealth/, www.fiskeridir.no and www.kartverket.no). Some of the production data cannot be made public because of privacy agreements. R code used for descriptive results, analysis, models diagnostics and creating figures is available (https://doi.org/10.5281/zenodo.4309632).
