# Supplementary material for: Factors associated with baseline mortality in Norwegian Atlantic salmon farming
Source: Sci Rep. 2021 Jul 19;11:14702. doi: 10.1038/s41598-021-93874-6 (PMC8289829; doi:10.1038/s41598-021-93874-6)
Supplement: Supplementary file 1 — Supplementary Information. [file 41598_2021_93874_MOESM1_ESM.pdf]

Factors associated with baseline mortality in Norwegian Atlantic salmon farming

Victor H.S. Oliveira[1*], Katharine R. Dean[1], Lars Qviller[1], Carsten Kirkeby[2] and Britt Bang Jensen[1]

[1]Section for Epidemiology, Norwegian Veterinary Institute, 1433 Ås, Norway.

[2]Department of Veterinary and Animal Sciences, Faculty of Health and Medical Sciences, University of Copenhagen, 1870 Frederiksberg, Denmark

*Author for correspondence: Victor H. S. Oliveira, 1433 Ås, Norway.

E-mail: victor.oliveira@vetinst.no (V.H.S. Oliveira)

Supplemental material

Supplementary Table S1: Results of the final fitted negative binomial regression model for the determinants of baseline mortality in farmed Atlantic salmon. Analysed data relates to 14,280 monthly records from 1,627 fish cohorts produced on 642 Norwegian farms between January 2014 and December 2019.

Variable	Category	Estimate	95% CI[a]	*p*-value
Intercept	–	-7.091	-7.305 – -6.878	–
Sea surface temperature	–	10.063	8.118 – 12.008	< 0.001
(Sea surface temperature)^2		9.656	7.955 – 11.364	
Sea surface salinity	–	0.037	0.032 – 0.042	< 0.001
Production zone	10 (Ref.)	–	–	< 0.001
	1	0.280	0.045 – 0.515	
	2	0.430	0.257 – 0.603	
	3	0.464	0.323 – 0.604	
	4	0.437	0.292 – 0.582	
	5	0.229	0.041 – 0.417	
	6	0.165	0.019 – 0.311	
	7	0.016	-0.170 – 0.201	
	8	0.092	-0.064 – 0.248	
	9	0.001	-0.152 – 0.153	
	11	0.317	0.134 – 0.500	
	12 and 13[c]	0.329	0.176 – 0.481	
Month of 1st stocking at sea	Sep (Ref.)	–	–	< 0.001
	Mar	0.210	0.126 – 0.295	
	Apr	0.263	0.195 – 0.330	
	May	0.252	0.182 – 0.321	
	Jun	0.220	0.127 – 0.313	
	Jul	0.305	0.225 – 0.385	
	Aug	0.025	-0.031 – 0.081	
	Oct	0.045	-0.031 – 0.122	
	Nov	0.174	0.052 – 0.299	
	Dec, Jan & Feb[c]	0.273	0.152 – 0.396	
Weight upon stocking at sea[b]	–	0.014	0.001 – 0.028	0.037
Fish weight	–	11.502	9.774 – 13.236	< 0.001
(Fish weight)^2	–	7.138	5.473 – 8.817	
H_2O_2 or medicinal treatments[d]	Not treated (Ref.)	–	–	< 0.001
	1 time per month	0.096	0.049 – 0.143	
	≥ 2 times per month[c]	0.159	0.090 – 0.230	
Non-medicinal treatments[e]	Not treated (Ref.)	–	–	< 0.001
	1 time per month	0.431	0.363 – 0.500	
	≥ 2 times per month[c]	0.593	0.479 – 0.708	

[a]CI =confidence interval.

[b]For every 50g change.
[c]Merged category due to few number of observations in one of them.
[d]Bath treatments using H_2O_2 or medicinal compounds, such as azamethiphos and pyrethroids.
[e]Removal of sea lice usually by flushing or brushing, warm water or freshwater baths.

Supplementary Table S2: Variables and their scales used for modelling monthly baseline mortality of farmed Atlantic salmon.

Variable	Scale	Explanation / unit
Fish counts	Discrete	No. of fish
Fish deaths	Discrete	No. of dead fish
Month counts	Discrete	No. of months at sea
Farm	Categorical	Farm identification
Sea surface temperature[a]	Continuous	Celsius
Sea surface salinity[a]	Continuous	Parts per thousand (‰)
Stocking month[a]	Categorical	Months of the year
Weight upon stocking at sea[a]	Continuous	Gram
Fish weight[a]	Continuous	Gram
Sea lice count[a]	Continuous	Mean female sea lice count
Production zone[a]	Categorical	Norwegian coast zones 1 to 13
Local biomass density[a]	Continuous	Summarized record of salmon biomass (i.e. number of fish multiplied by mean weight) calculated using data from neighbouring farms located up to 40 km seaway distance. See Jansen et al. (2012) for full details on LBD calculation
H_2O_2 or medicinal treatments[a]	Categorical	Bath treatments using H_2O_2 or medicinal compounds, such as azamethiphos and pyrethroids / 0, 1, or ≥ 2 times
Non-medicinal treatments[a]	Categorical	Removal of sea lice usually by flushing or brushing, warm water or freshwater baths / 0, 1, or ≥ 2 times

[a]Explanatory variables of the model.

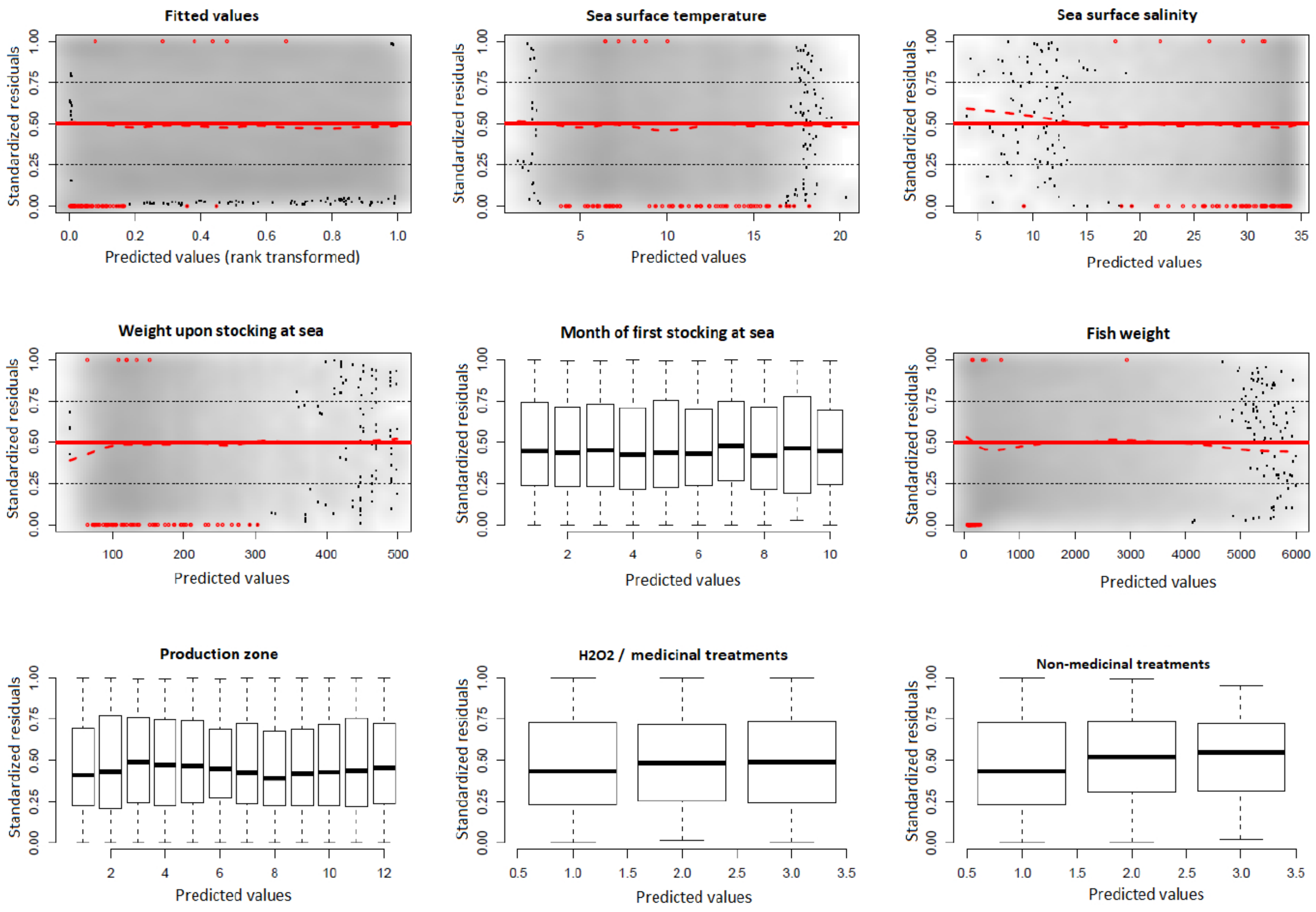

Supplementary Figure S1: Diagnostic plots of a fitted negative binominal regression model for the determinants of baseline mortality in farmed Atlantic salmon. This figure was generated using the DHARMa[86] package in R[78].

Reference

Hartig, F. DHARMa: Residual Diagnostics for Hierarchical (Multi-Level / Mixed) Regression Models. *CRAN* https://cran.r-project.org/web/packages/DHARMa/DHARMa.pdf (2020).

Jansen, P. A. *et al.* Sea lice as a density-dependent constraint to salmonid farming. *Proc. R. Soc. B Biol. Sci.* **279**, 2330–2338 (2012).

R Core Team. R: A language and environment for statistical computing. R Foundation for Statistical Computing, Vienna, Austria. (2020).
